# Modeling and subtleties of K-Ras and Calmodulin interaction

**DOI:** 10.1371/journal.pcbi.1006552

**Published:** 2018-10-30

**Authors:** Eduardo Garrido, Juan Lázaro, Montserrat Jaumot, Neus Agell, Jaime Rubio-Martinez

**Affiliations:** 1 Department of Materials Science and Physical Chemistry, Universitat de Barcelona, Institut de Recerca en Química Teòrica i Computacional (IQTCUB), Barcelona, Spain; 2 Department de Biomedicina, Facultat de Medicina i Ciències de la Salut, Universitat de Barcelona, IDIBAPS, Barcelona, Spain; Fox Chase Cancer Center, UNITED STATES

## Abstract

K-Ras, one of the most common small GTPases of the cell, still presents many riddles, despite the intense efforts to unveil its mysteries. Such is the case of its interaction with Calmodulin, a small acidic protein known for its role as a calcium ion sensor. Although the interaction between these two proteins and its biological implications have been widely studied, a model of their interaction has not been performed. In the present work we analyse this intriguing interaction by computational means. To do so, both conventional molecular dynamics and scaled molecular dynamics have been used. Our simulations suggest a model in which Calmodulin would interact with both the hypervariable region and the globular domain of K-Ras, using a lobe to interact with each of them. According to the presented model, the interface of helixes α4 and α5 of the globular domain of K-Ras would be relevant for the interaction with a lobe of Calmodulin. These results were also obtained when bringing the proteins together in a step wise manner with the umbrella sampling methodology. The computational results have been validated using SPR to determine the relevance of certain residues. Our results demonstrate that, when mutating residues of the α4-α5 interface described to be relevant for the interaction with Calmodulin, the interaction of the globular domain of K-Ras with Calmodulin diminishes. However, it is to be considered that our simulations indicate that the bulk of the interaction would fall on the hypervariable region of K-Ras, as many more interactions are identified in said region. All in all our simulations present a suitable model in which K-Ras could interact with Calmodulin at membrane level using both its globular domain and its hypervariable region to stablish an interaction that leads to an altered signalling.

## Author summary

K-Ras is one of the most mutated oncogenes in human cancer. Although several studies validate K-Ras protein as good candidate for direct therapeutic targeting, pharmacologic targeting has not been successful. During the last years increasing evidences demonstrate that oncogenic K-Ras activity can be modulated in vivo by dimerization, nanoclustering at the plasma membrane or interaction with non-effector proteins, consequently opening new therapeutic strategies. We have previously demonstrated that Calmodulin, an ubiquitous Ca2+-binding protein, is one of this K-Ras interacting proteins and that it negatively modulates K-Ras signaling. Although experimental data were available showing the relevant regions for this interaction, a model of K-Ras and Calmodulin interaction was missing. In the present work by using different computational modeling techniques we obtained a model for this interaction that agrees with the experimental data. We believe the present model will help to better understand K-Ras regulation, and to design new inhibitors. For instance, base on our model, we can predict that the interaction can take place at the plasma membrane, and that since the surface of K-Ras that interact with Calmodulin is the same that it uses for dimerization, that Calmodulin could be inhibiting K-Ras dimerization.

## Introduction

Ras proteins are well-known small GTPases involved in the regulation of key signal transduction pathways. Cycling from the inactive (GDP-bound) to the active (GTP-bound) state, they faithfully respond to extracellular signals due to their tight regulation by GTP-exchange factors (GEFs) and GTPase activating proteins (GAPs). In the GTP-bound form, two regions of the protein change conformation (switch I and II domains) allowing its binding with different effector proteins and consequently the activation of diverse signal transduction pathways. Among those, the best characterized are the RAF1/MEK/ERK and the phosphatidylinositol-3-kinase (PI3K)/AKT [1], which are known to regulate proliferation, differentiation and survival. Activating point mutations render Ras proteins that are always found in the GTP-bound state independently of the extracellular signals and are crucial steps in the development of the vast majority of cancers [2]. Ras genes were the first oncogenes identified in human cancer cells, and nowadays they are well established as the most frequently mutated oncogenes in human cancer [3]. Three different genes code for a total of four different Ras isoforms named H-Ras, N-Ras, K-RasA and K-RasB (herein after referred to as K-Ras). K-Ras is the most frequently mutated oncogene in solid tumors and its oncogenic mutations occur mostly in pancreatic ductal adenocarcinomas (95%), colon (40%) and adenocarcinomas of the lung (35%) [3–5]. Although they all have a highly conserved globular domain (from residue 1 to 165) containing the guanosine nucleotide and effector binding sites (Switch I and Switch II), the last C-terminal residues of Ras proteins, named the hypervariable region (HVR), which contains the membrane targeting signals, are not conserved among the different isoforms. H- and N-Ras achieve high-affinity hydrophobic membrane binding mainly through lipid modifications. By contrast, K-Ras has, adjacent to the farnesylated cysteine Cys185, a stretch of lysine residues—known as the polybasic domain—that promotes an electrostatic interaction with the negatively charged phospholipids [6,7], which confines K-Ras almost entirely to non-raft microdomains within the plasma membrane [8].

The different membrane anchors interact with lipids and proteins of the plasma membrane and, together with the hypervariable region (HVR), drive the Ras isoforms into spatially and structurally distinct nanodomains, of which each then contains a cluster of molecules (nanocluster) [9–11]. Importantly, the nanodomains that are occupied by the three isoforms of Ras do not show any overlap. Furthermore, not only are the different Ras isoforms laterally segregated, but inactive GDP-loaded Ras occupies nanodomains that are spatially distinct from those occupied by the active GTP-loaded form. Formation of these nanoclusters is essential for activation of mitogen-activated protein kinases (MAPKs), because they constitute exclusive sites in the plasma membrane for Raf-1 recruitment and ERK activation [12–14].

Because oncogenic mutations of K-RAS give rise to an always GTP-bound protein that constitutively binds to effectors, positive or negative physiologic regulation of oncogenic K-RAS was not initially expected. In recent years, interaction of K-Ras with proteins, which are not effectors but which may function as allosteric regulators or scaffolds, have been described and some proved to be crucial to fully display K-RAS oncogenic phenotype. Galectin-3 [15], calmodulin (CaM) [16], phosphodiesterase δ [17,18], nucleophosmin, nucleolin [19] and heterogeneous nuclear ribonucleoprotein A2/B1 (hnRNPA2/B1) [20] have been shown to interact with K-Ras and modulate its downstream signaling. The mechanism by which these proteins modulate K-Ras signaling is diverse: phosphodiesterase δ by binding to the farnesyl group facilitates the diffusion of K-Ras from endomembrane to the cytoplasm, ultimately favoring its correct relocalization to the plasma membrane and consequently enhances its signaling [18]; Galectin-3 regulates K-Ras nanoclustering at the plasma membrane and also enhances its signaling [15]; and, hnRNPA2/B1 favors the interaction of K-Ras with PI3K [20]. In contrast, while K-Ras interaction with CaM has been known for many years, there is still some controversy regarding the consequences of this interaction. Our group demonstrated that CaM interaction with K-Ras inhibits K-Ras signaling to Raf/MEK/ERK [16] and inhibits K-Ras phosphorylation at Ser181 in the HVR [21]. Interestingly, CaM also binds to PI3K enhancing its activity [22], and the existence of a complex containing K-Ras, CaM and PI3K has been proposed [23].

CaM is a small (148 amino acids) and well conserved Ca^2+^-binding protein [24]. The crystal structure of CaM in the Ca^2+^-bound form shows a dumbbell-shaped molecule with two globular domains arranged in a trans configuration. These domains are connected by a long extended central α-helix, the middle portion of which is highly mobile and acts as a flexible tether. Each domain consists of two helix-loop-helix motifs (EF hands), with each binding one molecule of Ca^2+^. Ca^2+^ binding changes the orientation of the two EF hands of each domain, inducing the appearance of hydrophobic patches that interact with proteins known as CaM-binding proteins (CaMBPs) [25]. Binding of CaM to CaMBPs modulates the function of these proteins and, in consequence, affects many aspects of cell regulation. The carboxyl-terminal lobe binds Ca^2+^ with high affinity (Kd 10^−7^ M), whereas the amino-terminal lobe binds it with lower affinity (Kd 10^−6^ M). The fact that the Kd values fall within the range of intracellular Ca^2+^ concentration exhibited for most cells (10^−7^–10^−6^ M) makes it a good sensor for changes in Ca^2+^ intracellular levels [26–28].

The CaM binding domain of some of the CaMBPs with high affinity for CaM (nM range) consists of a 20-amino acid sequence that has an amphipathic α-helix conformation [29]. CaM binding domains with lower affinity for CaM (μM range) have also been described [30]. Some proteins like MARCKS and CAP-23/NAP-22 use the myristoyl group to interact with CaM [31,32]. As well as K-Ras, diverse Ras superfamily GTPases like Kir/Gem [33], Ric [34], Rin [35], Rab3A [36], and RalA [37] have been shown to bind to CaM. Biochemical data indicate that at least two different regions in the K-Ras molecule are important for K-Ras/CaM interaction: the hypervariable region and the α-helix between amino acids 151 and 166 [38]. Within the hypervariable region, both the hydrophobic farnesyl group and the positive-charged amino acids were essential for the interaction between K-Ras and CaM. Consistently, K-Ras S181D mutant, which mimics phosphorylation of Ser-181 of K-Ras, also completely abolished binding to CaM. Although the presence of the farnesyl group increases the affinity of purified K-Ras to CaM, full length non-farnesylated K-Ras still has micromolar affinity for CaM [39]. Accordingly to the above mentioned, the NMR data of this complex show that the N-terminal lobe of CaM interacts with the globular domain of K-Ras and the C-terminal lobe of CaM interacts with the HVR [40].

But controversial data exist regarding how CaM interaction with K-Ras could modulate K-Ras activity. While some data indicate that CaM could extract K-Ras from membranes *in vitro*, most probably by interacting with the farnesyl group [41,42], it is not clear if *in vivo* this hydrophobic group would always be available for CaM to interact with. In fact, our group has demonstrated that K-Ras and CaM colocalize mainly in the plasma membrane, suggesting that *in vivo* interaction does not directly lead to K-Ras internalization [38]. CaM could be modulating interaction of K-Ras within the plasma membrane, with effectors, scaffolds or with different lipids, ultimately regulating K-Ras signaling from the plasma membrane. Thus, modelling of K-Ras/CaM interaction is important to decipher the cellular role of this interaction. To mimic the situation of K-Ras bound to the membrane, thus with farnesyl group hindered between the phosphoslipids, we aimed to model the interaction between a full length non-farnesyated K-Ras and CaM.

CaM and K-Ras have been widely studied computationally [43,44]; in fact, CaM is one of the most studied proteins with molecular dynamics (MD) due to its high degree of flexibility. These systems have also been joined to a certain degree [45], but up to date no simulations of the whole proteins have been performed. Thus, we decided to carry on conventional MD (cMD) on a system with both proteins in order to determine which the details of the interaction are.

Furthermore, in order to increase the exploration of the conformational space of the K-Ras/CaM system, scaled MD (sMD) a recently developed methodology that proved to be effective to sample wider conformational areas faster than cMD [46], was used.

## Results

### K-Ras and CaM tend to interact in both cMD and sMD

In order to computationally study the interaction between K-Ras and CaM, a system with both proteins had to be prepared. Since NMR experimental data regarding the interaction between these two proteins has already been published [40], we decided to mimic the experimental settings: oncogenic K-Ras (G12D mutation) full-length without post-translational modifications paired with holo-CaM.

**Fig 1:**
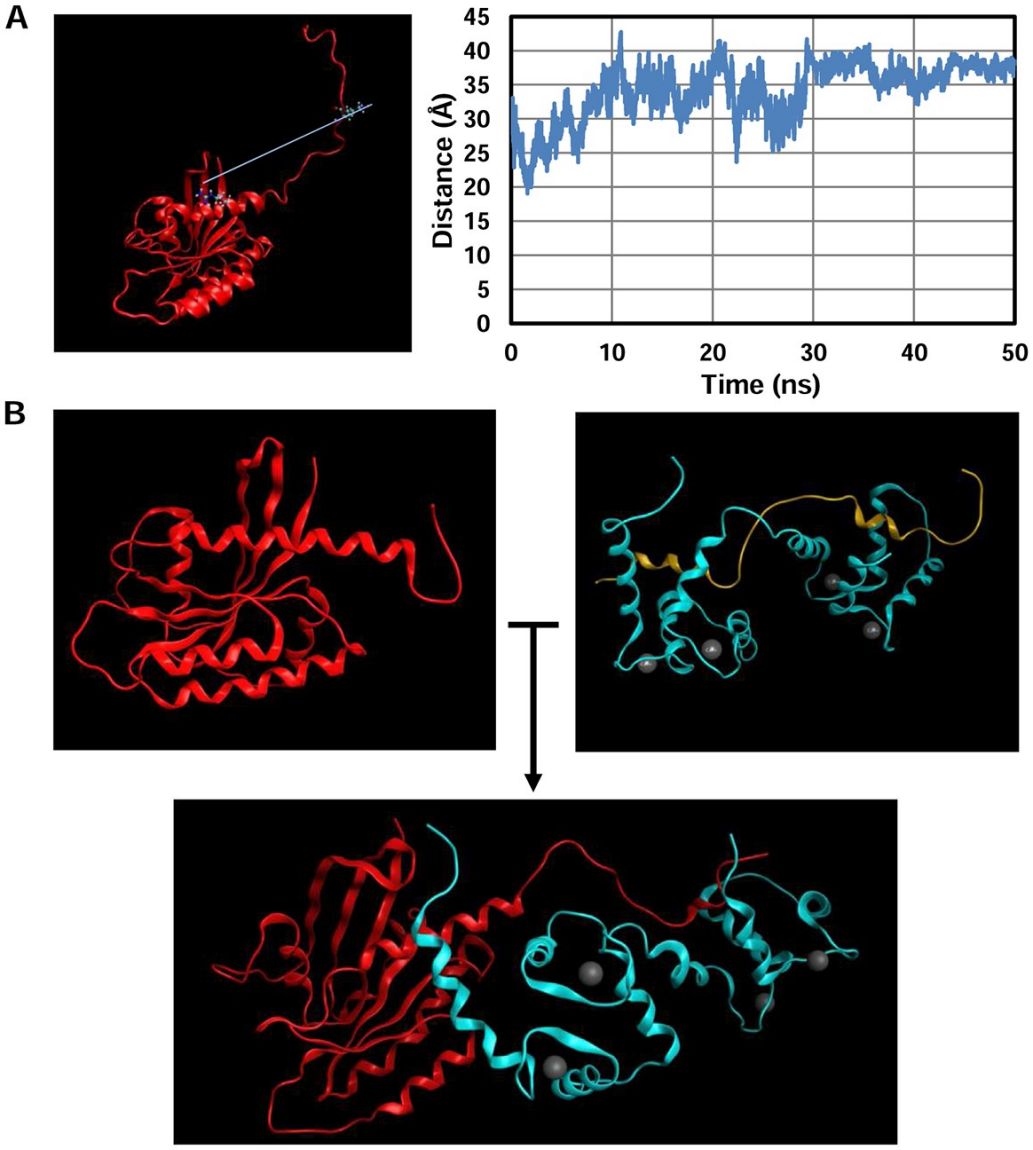
K-Ras/CaM system assembling. A) A system comprising active K-Ras (PDB code 4DSN) with its HVR region extended was prepared (left image) and 50 ns of cMD simulations were performed. The distance between residues 161 and 178 was calculated and depicted in the right graph. B) The peptide from the structure with PDB code 2MGU (light yellow) was replaced with the HVR of K-Ras with the Modeller software and reoriented within the lobes of CaM (light blue). Afterwards, the globular domain of K-Ras (in red) was jointed to the HVR.

Prior to a simulation between the proteins, a system composed of GTP-bound K-Ras with a fully extended HVR was prepared. This system was used to determine whether the HVR could be found in an extended conformation in several frames or if it would be mostly bent to interact with the globular domain. Fifty nanoseconds of cMD were performed and the provided trajectories were analyzed by measuring the distance between residues 161 (from the α-helix 5) and 178 (from the HVR). The HVR presented an extended conformation most of the time, showing great motility (Fig 1A). Interestingly, other groups have seen similar behavior when simulating K-Ras in its active state, reporting that the HVR does not significantly interact with the globular domain [47].

Since the binding of these two proteins does not seem to be mediated by the common binding mechanism of CaM (where it wraps its lobes around a single structure, such as an α-helix), we decided to use the structure of CaM with PDB code 2MGU. This structure presents its lobes rather extended, which could fit with a model in which the N-lobe of CaM interacts with the globular domain of K-Ras and the C-lobe interacts with the HVR. The peptide present in the structure was replaced by the HVR of K-Ras with Modeller, and subsequently rotated to fit the model (see Methods for more details). Last, the globular domain of K-Ras was attached to obtain the system with both proteins (Fig 1B).

**Fig 2:**
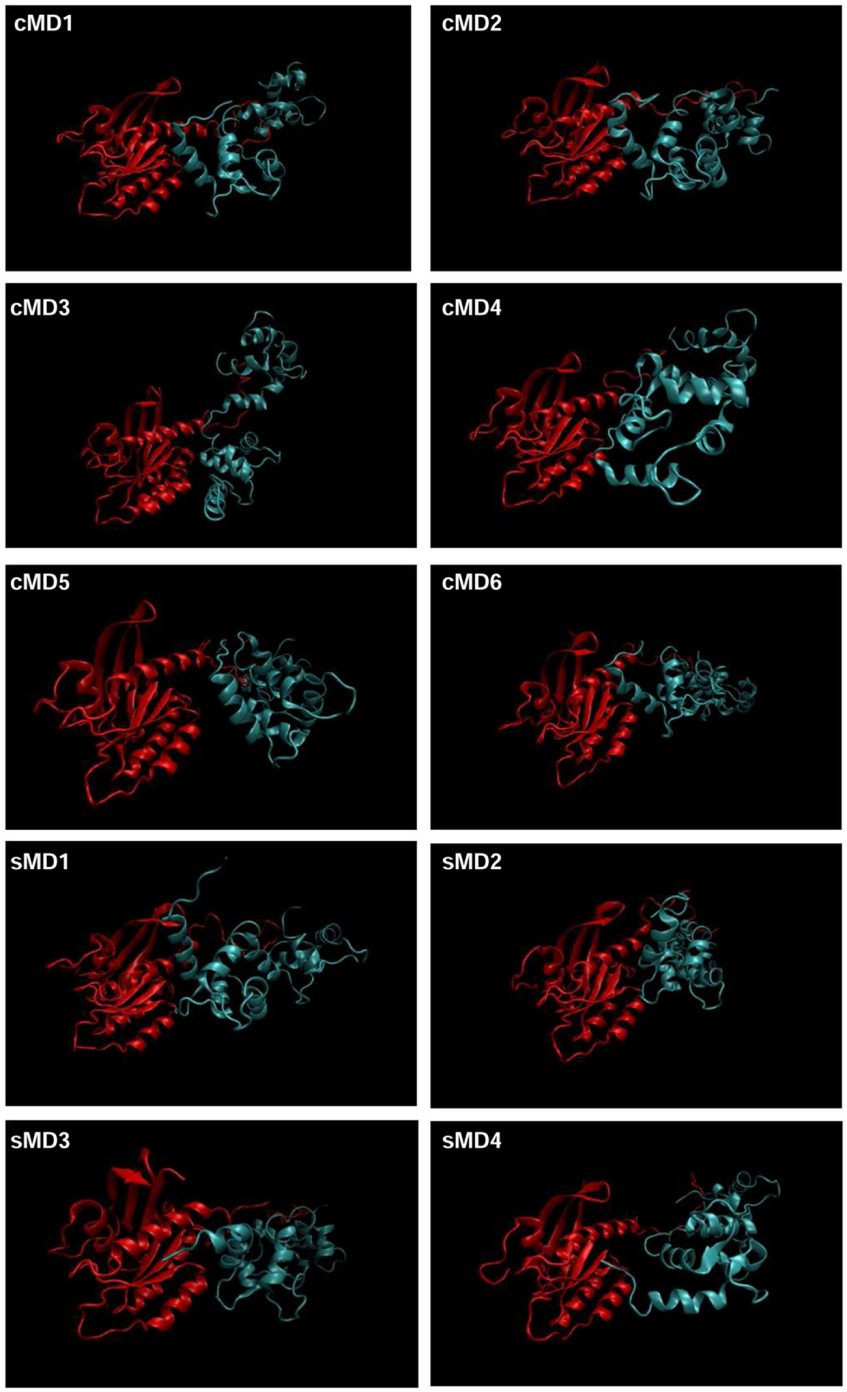
Snapshots of the K-Ras/CaM simulations. Last structure for each MD were obatined. All simulations except for cMD 4 maintained a stable interaction between both proteins. K-Ras is shown in red, while CaM is represented in blue.

Once the system was prepared, a total of 6 cMD and 4 sMD simulations were carried out, each of them with a total length of 50 ns. The trajectories were visually analyzed in order to determine which simulations had stablished a proper interaction between the two proteins, and in which K-Ras/CaM had fallen apart. Interestingly, in 9 out of 10 simulations the proteins interacted throughout most of the simulation length, even with the additional energy boost of the sMD simulations (Fig 2). Furthermore, the N-Terminal domain of CaM remained close to the α-helix 5 of K-Ras in most of the simulations. The end of the HVR maintained a close contact with the C-Terminal lobe of CaM, while the polybasic domain of K-Ras interacts with the linker region of CaM.

**Fig 3:**
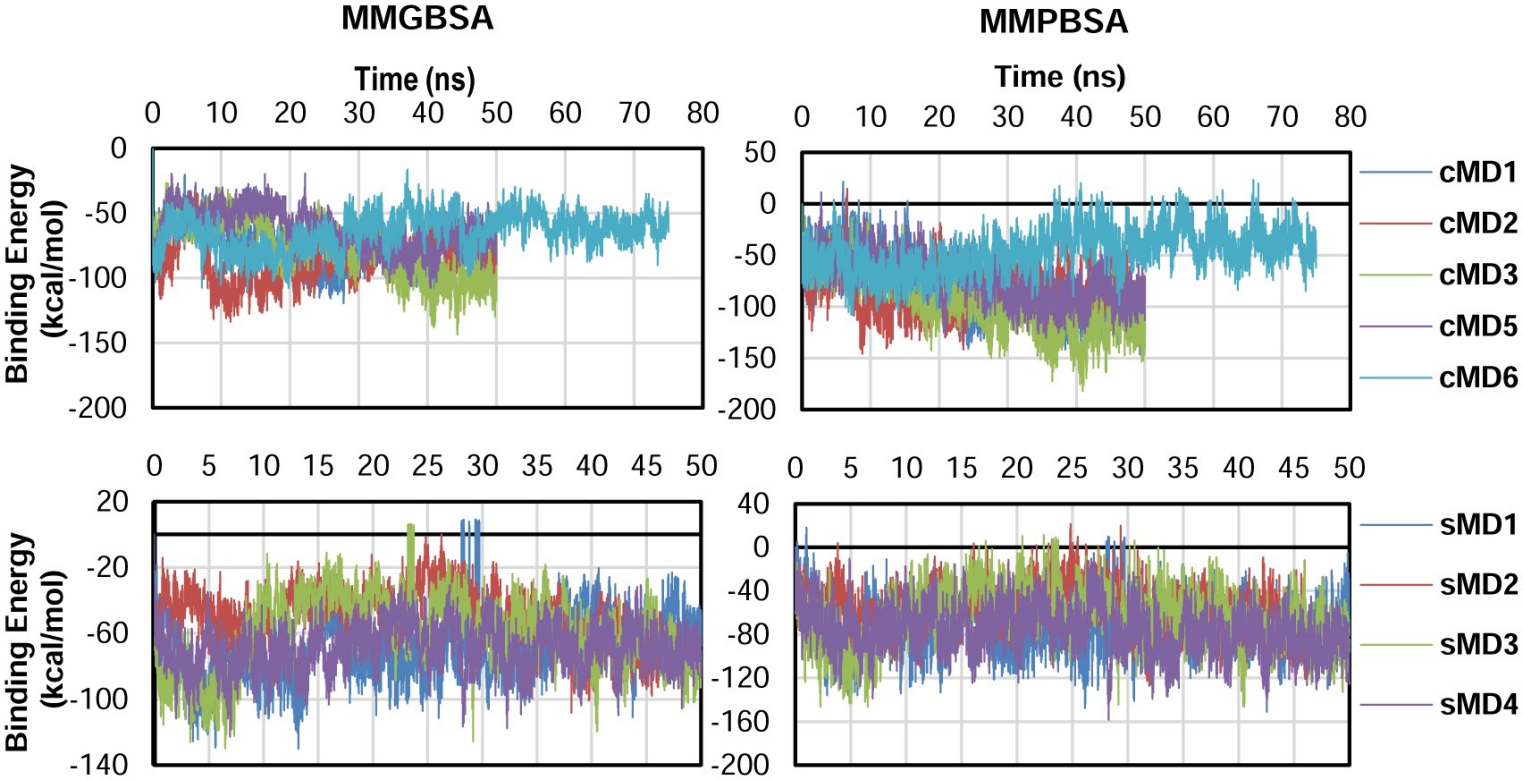
Energy profiles of the simulations either with MMGBSA (left) or MMPBSA (right). Energy profiles of the cMD simulations (top). Energy profiles of the sMD simulations (bottom).

The energy of the system was determined by performing a MMPB/GBSA analysis. The dynamics were considered stable if the last 5 ns did not present significant deviations. If any of the simulations were not stable enough, they were extended until stability was reach. The energy profile was similar for PB and GB. The interaction presented between -60 and -100 kcal/mol for GB and between -40 and -120 kcal/mol for PB both for cMD and sMD (Fig 3).

### The simulations used fit the available experimental data

**Fig 4:**
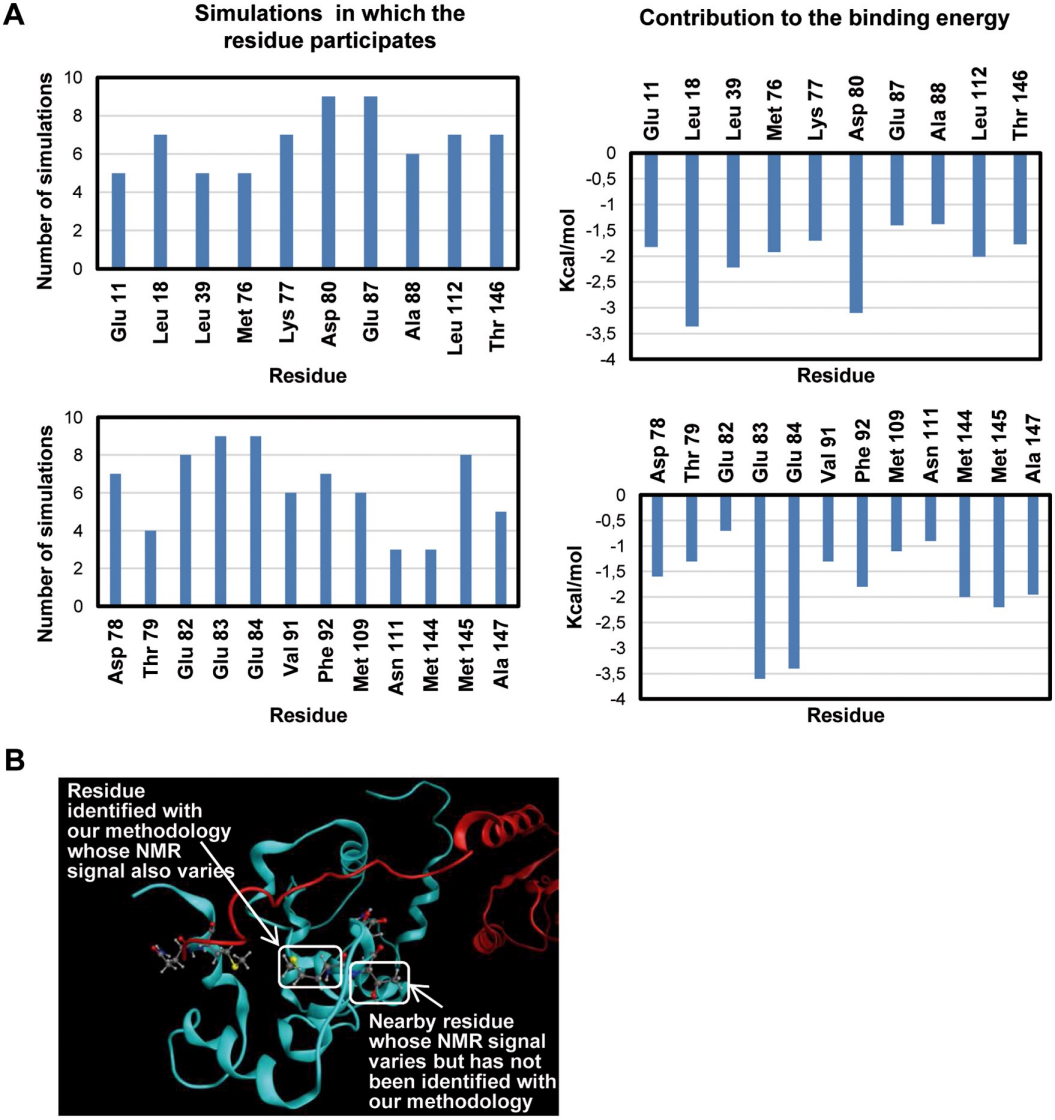
Residues of CaM that participate in the interaction. A) The energies (right) and the number of times the matching residues appeared in the simulations (left) of the residues that surpassed the established thresholds. Upper graphs correspond to residues that match the experimental data, lower graphs to the residues that did not match experimental data. B) Possible origin of mismatch between theoretical and experimental data.

The last ns of each simulation were used to calculate the contribution of each residue to the binding energy through a “per residue” analysis. The residues of CaM were studied in order to find matches with the experimental data available. Two thresholds were imposed to consider a residue as actively participating in the interaction between K-Ras and CaM: the first was a requisite of at least -0.7 kcal/mol of average contribution to the binding, whereas the second was its presence in at least 3 of the simulations. Up to twelve residues matched with the experimental data available, many of which are negatively charged residues (78 to 84) that can interact with the polybasic domain of K-Ras (Fig 4A). Intriguingly, certain residues of CaM whose surroundings are modified when interacting with K-Ras (experimentally) do not present a significant implication in the interaction between both proteins in the simulations (Fig 4A). The presence of changes in nearby residues when binding to other proteins can explain why there are NMR shifts assigned to those residues while no energy contribution is seen in our simulations (Fig 4B). With all things considered, the model can be considered robust enough to analyze the residues of K-Ras that participate in the interaction, some of which have not been described yet.

### Both the globular domain and the HVR of K-Ras present interactions with CaM

**Fig 5:**
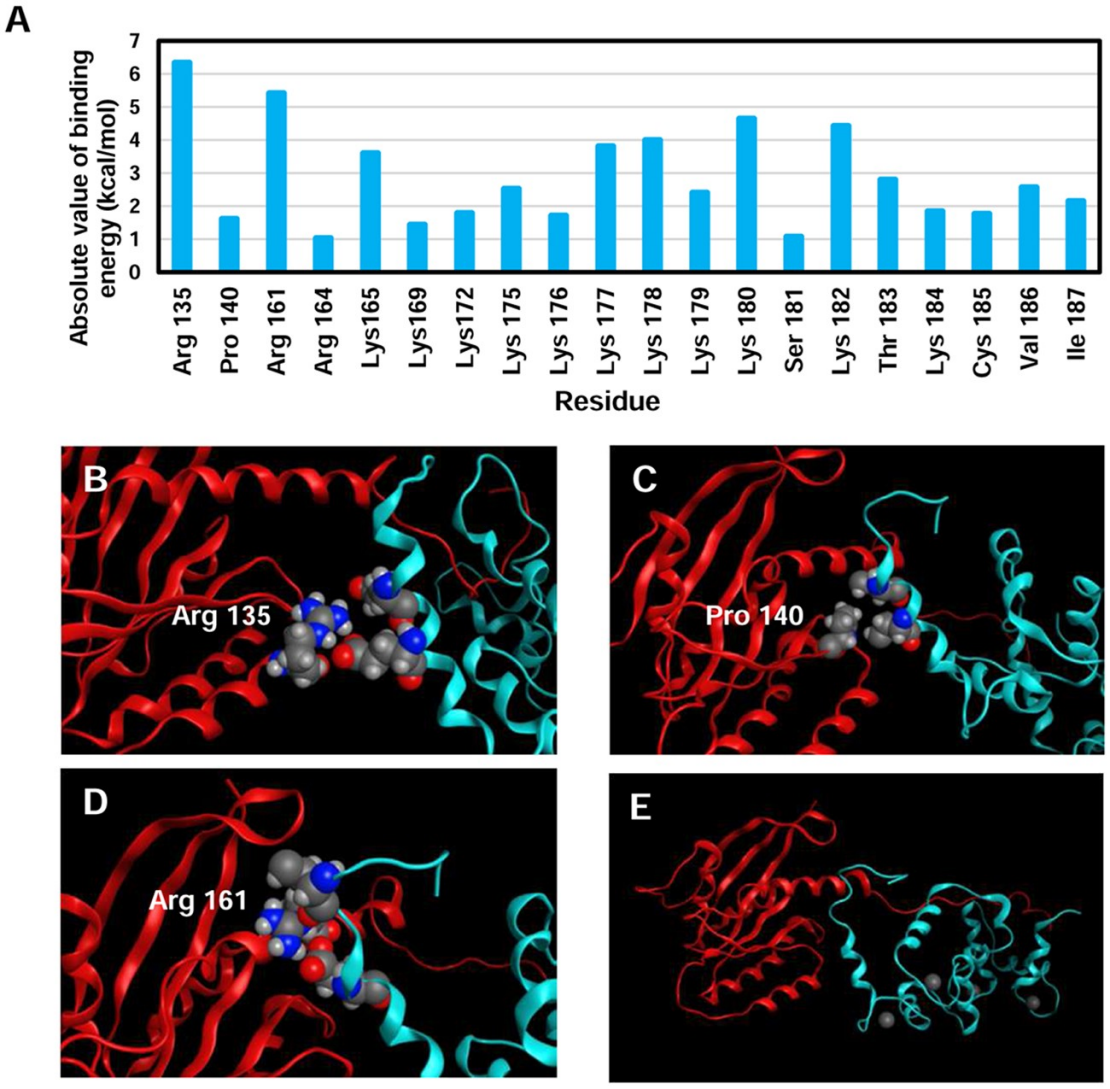
K-Ras residues relevant for the interaction. A) Residues that surpassed the thresholds imposed. B-E) Snapshots reflecting the interaction of the globular domain (B-D) and the HVR (E) with CaM.

After analyzing the residues of CaM, we focused on the residues of K-Ras relevant for the interaction. A threshold of -1 kcal/mol of average was imposed to the residues that participated in the interaction. Also, their participation had to be present in at least 5 simulations. In concordance with the experimental data, most of the residues responsible for the interaction were found within the HVR. However, 5 residues were identified in the globular domain. Furthermore, most of them presented energy values below -3.5 kcal/mol, being arginine 135 the most significant residue in terms of average energy (Fig 5A).

When visualized, the simulations revealed that the selected residues of the globular domain were, in fact, closely interacting with CaM. The arginines from the α –helix 5 formed hydrogen bonds with the EF hand of the N-Terminal domain, while arginine 135 and proline 140 interact with one of the four α helixes present in the N-Terminal lobe of CaM (Fig 5B–5E).

**Fig 6:**
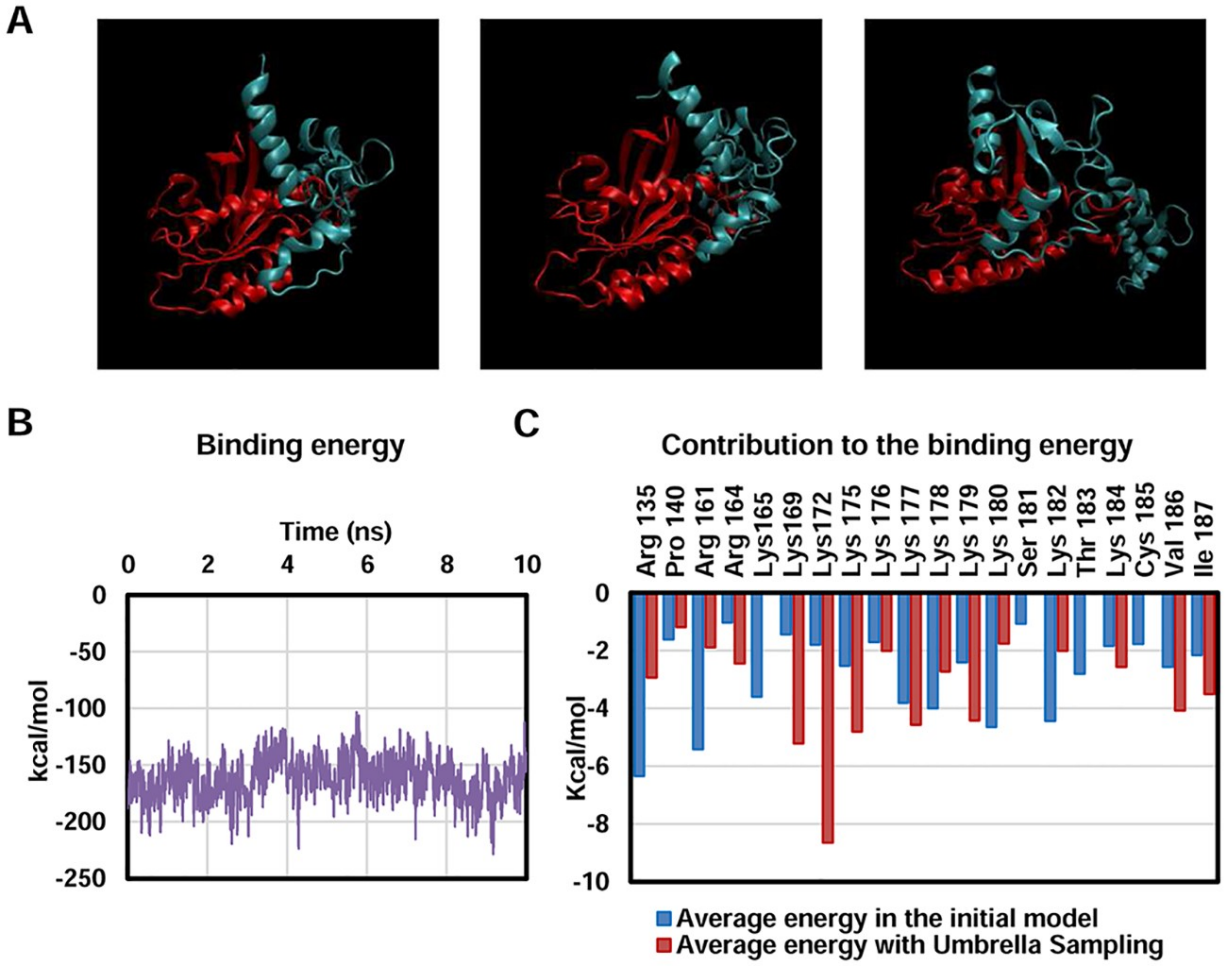
Validation of the residues of K-Ras participating in the interaction with umbrella sampling. A) Snapshots of the cMD simulations initiated with the structures of the umbrella sampling. B) Representative MMGBSA of the simulations. C) Comparison of the residues that surpassed the threshold either in the initial model (both cMD and sMD) or in the umbrella sampling.

To further confirm the results obtained with the performed simulations, a different methodology was used: the umbrella sampling. This kind of simulation allows a more progressive scenario for the proteins to adapt, as more time is given to position them nearer. To perform this simulation, a restriction was added to maintain the mass center of the α-helix 5 of K-Ras and the mass center of the N-Terminal lobe of CaM at a prefixed distance. Then, the restricted groups were slowly approached, at a rate of 0.5 Å per step, remaining for 0.5 ns at each distance before closing the gap between them. The initial distance was set at 20 Å, while the last step was set at 5 Å. Once the simulations were performed, structures from the US with the mass centers maintained at 5, 6, 7 and 8 Å of distance were obtained and 10 ns of cMD were calculated. All these simulations presented high interaction between K-Ras and CaM, with the N-Terminal lobe of CaM wrapping the α-helix 5 of K-Ras and the HVR embedded in the C-Terminal lobe of CaM (Fig 6A).

A MMGBSA analysis was also performed to determine the binding energy of the proteins and analyze the stability of such interaction (Fig 6B). Values around -150 kcal/mol were obtained for all four simulations, exceeding the values seen in simple cMD or sMD (whose values were around -100 Kcal/mol). A “per residue” analysis was also performed so as to determine if the residues described as relevant with the previous methodology were still actively participating in the binding. Since these residues had more time to accommodate and orientate in a favorable angle for the interaction, only those residues actively interacting in the four simulations with an average energy below -1kcal/mol were selected. Even though according to the US simulations some residues selected with the initial model were not relevant for the interaction, most of them matched. Furthermore, only one of the studied residues of the globular domain did not surpass the thresholds, which backs up the idea that the globular domain is playing a part in the interaction (Fig 6C).

Taking into account all the data provided by the simulations performed, it seems the globular domain interacts with CaM, specifically through residues R135, P140, R161, R164 and, to a minor extent, K165.

### Mutation of specific residues of the globular domain of K-Ras diminish its affinity with CaM confirming MD data

**Fig 7:**
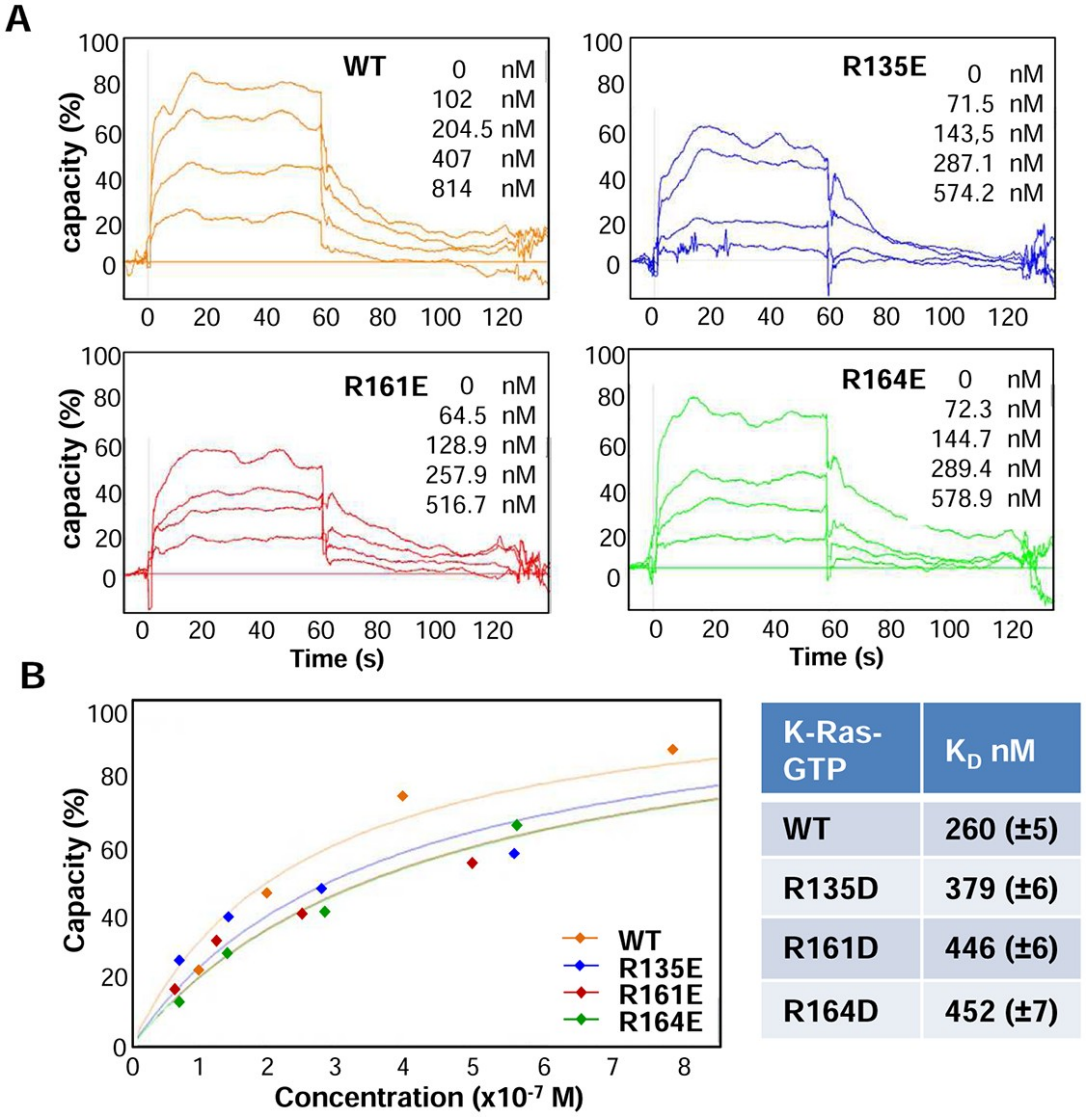
Experimental validation of the results of the simulations. A) Sensograms of a representative SPR experiment to determine the affinity between the indicated mutants of K-Ras and CaM. B) Image of the fitting of the curves used to calculate de K_D_ using the affinity method and table with calculated K_D_ from the data shown in A).

With the purpose of verifying the obtained results with experimental data, three mutants of the globular domain (1–166 aa) were obtained through point mutation. The mutants were designed according to the results of the simulations: R135E, R161E and R164E. The corresponding GST-K-Ras mutants were expressed in bacteria, affinity purified and then its binding to CaM determined by Surface Plasmon Resonance (SPR). Biotinilated CaM was attached to a chip with streptavidin and the GST-tagged globular domain of K-Ras (either wild-type or mutant) was injected as an analyte. A control flow cell with no CaM, was also injected with the globular domain as a blank. To discard that the binding was due to the GST tag, recombinant GST was injected in all flow cells and no binding was observed. The injection of the globular domain of K-Ras led to an increase in the Resonance Units (RU) of the flow cells with CaM, which stemmed from the binding of the injected protein to CaM. The mutants also showed binding with CaM, but to a lower extent. An affinity study was performed to determine the K_D_, and the results reflected that the wild-type globular domain presented a lower K_D_ than any mutant. All the mutations led to an increase in the K_D_, that is, in a reduction of the affinity with CaM (Fig 7). Thus, our experimental data support the results obtained through computational simulations, where these 3 residues were identified as key players in the interaction of the globular domain of K-Ras with CaM.

## Discussion

Even though it has been almost twenty years since the discovery of the interaction between K-Ras and CaM [16], its subtleties have remained elusive. In the present work, thanks to the use of computational aided techniques, we have shed some light upon this interesting matter.

Since these proteins have never been simulated computationally, we looked for a system with validated experimental results to be able to contrast the data generated. Thus, we used full-length K-Ras without its farnesylation, as this system had already been experimentally studied with NMR [40]. Even though we are aware of the relevance of the post-translational modifications of K-Ras for the interaction with CaM, it’s unlikely that CaM can initially interact with the farnesyl group, as it will be attached to the plasma membrane. Thus, our simulations could mimic a situation in which the farnesyl group is not available for the interaction, as it would be hidden within the membrane. We used CaM with an extended conformation, with each lobe interacting with different domains of K-Ras, rather than wrapping around an specific region, similar to the interaction with other proteins in which the lobes of CaM interact with different regions [25]. It could be observed that the interaction between the proteins was stable, as it was maintained throughout most of the simulations. Furthermore, several residues of CaM matched the experimental data, despite the motility present in the system and the energy boosts provided in the sMD simulations.

Our group had previously described the participation of the globular domain and specially α-helix 5 of K-Ras in the interaction with CaM [38]. In the present work, we have taken another step forward to model this interaction and have confirmed and identified new residues of the globular domain that are implicated in said interaction. Arginine 161, arginine 164 (α-helix 5) and, to a minor extent, arginine 135 (α-helix 4) seem to be responsible of the interaction with the N-terminal lobe of CaM, since single point mutations in any of those residues lead to an increase in the experimental values of K_D_ between the globular domain of K-Ras and CaM. Interestingly, previous publications have highlighted the relevance of residue 135 in Ras signaling, as mutations in this residue led to enhanced binding with C-Raf RBD [48]. This phenomena may be explained by the diminished interaction with CaM, as the binding of this protein to K-Ras is known to diminish MAPK pathway signaling [16]. While we previously showed that the K-Ras switch II mutant, R68D/R73D, had a compromised interaction with CaM [38], our present model does not predict direct interactions of these two arginines with CaM. The most plausible explanation is that the substitution of the two positive residues by negative ones induces a conformational change in K-Ras, and indirectly, an increase in the negative charge density in the surface of CaM interaction that prevents the binding with this acidic protein.

As for the HVR, the simulations we have performed here proven to fit the available data. The highly negatively charged linker region of CaM is attracted to the polylysine domain of K-Ras, where they interact through electrostatic couplings. This fact has already been described experimentally by other groups [39]. As for the last C-Terminal residues of K-Ras, they are embedded by the C-Terminal lobe of CaM. However, it must be considered that this interaction may vary after the post-translational modifications, as the–AAX residues are removed and the farnesyl group is attached. Interestingly, in most of the simulations (six out of nine) cysteine 185 is not embedded within the C-terminal lobe of CaM, which would fit with a model in which the farnesyl group of this residue would be attached to the plasma membrane.

Our simulations can help to understand why the phosphorylation of K-Ras leads to the abrogation of this interaction with CaM [49]. As shown in the performed MD, the polybasic region of K-Ras plays an important role in the interaction with CaM, creating electrostatic interactions with the acidic linker region. Thus, the addition of a phosphate group, highly negatively charged, is bound to have a negative impact on the K-Ras/CaM interaction.

Our model may also provide one of the reasons why CaM does only interact with K-Ras when bound to GTP (its active state) [16]. Since the α-helixes of K-Ras are oriented towards the membrane when bound to GDP [50], the N-Terminal lobe of CaM would not be able to reach its interaction zone with the globular domain of K-Ras, as it would be covered by the PM. When active, K-Ras would expose its α-helix 4 and 5, giving the N-Terminal lobe of CaM a chance to interact with it. However, lack of interaction of full-length GDP-loaded K-Ras has also been described in the absence of lipid membranes. In this case the proposed autoinhibitory effect of the HVR could prevent CaM binding [44]: the globular domain would be inaccessible for CaM due to its binding with the HVR when K-Ras is in its GDP bound state, but would become reachable when GTP is loaded and the HVR is released. In fact, our simulations support the idea that, when bound to GTP, the HVR of K-Ras is not stably interacting with the globular domain. The study we performed of the dynamism of the HVR revealed that, even though there are some interactions between these groups, they are neither stable nor prolonged through much more than a few nanoseconds, thus giving to CaM the opportunity to interact with the HVR. However, it must be considered that experimental data show that CaM fails to interact with the purified inactive globular domain of K-Ras [16], so, despite our model being able to provide some explanations, a few details remain elusive.

**Fig 8:**
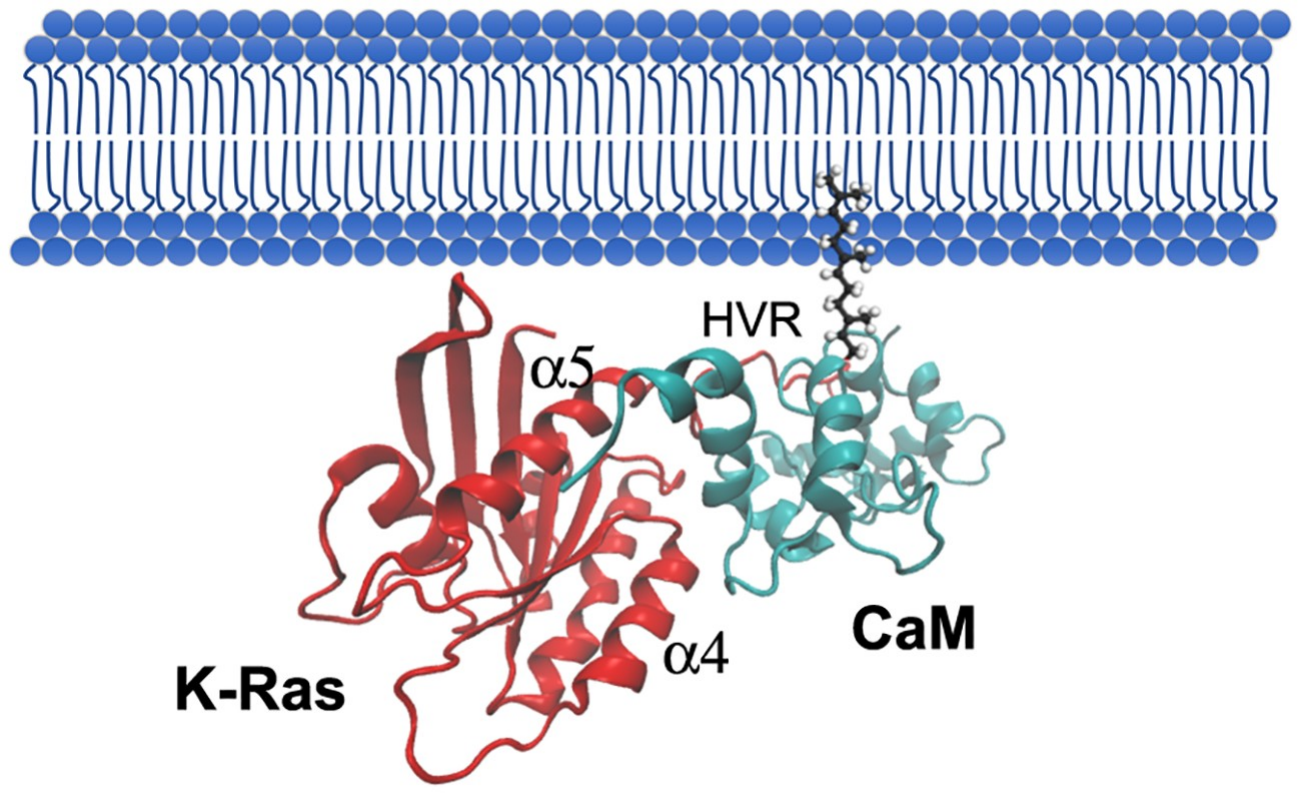
Proposed model of union of K-Ras with CaM in the presence of plasma membrane. CaM is depicted in light blue, while K-Ras is shown in red and the phospholipids in dark blue. The farnesyl group is attached to the plasma membrane while both proteins interact. The α5 and α4 helixes and the HVR of K-Ras are indicated.

Beyond the ins and outs of the interaction, the biological significance of such binding is becoming more interesting day after day. Although other interaction models between CaM and K-Ras may be feasible, especially with cytosolic K-Ras, our simulations would support a model in which K-Ras and CaM would interact at membrane level without indispensably inducing an extraction of K-Ras from the membrane (Fig 8), a fact that has been previously described by our group [38], and lately supported by recent publications that demonstrate that CaM can bind to K-Ras even when attached to nanodiscs emulating diverse types of PM [45]. In fact, CaM interaction with K-Ras may be modulating K-Ras clustering and signaling from the PM. For instance, CaM is thought to form a ternary complex with K-Ras and PI3K, which would enhance K-Ras signaling through AKT signaling while diminish it through Raf [23]. Our simulations provide interesting data suggesting that, while keeping one of its lobes interacting with K-Ras (probably the C-Terminal due to the interaction of the linker region with the polybasic domain), CaM could use its other lobe to interact with PI3K.

Also regarding K-Ras signaling, several authors have described the relevance of K-Ras dimerization in the activation of downstream effectors. While dimerization through α-helix 1 and β sheets 1/2 would inhibit the binding of effectors such as Raf or PI3K, due to the overlapping interaction surfaces, dimerization of K-Ras using the α-helixes 3/4/5 and β sheet 2 has been proposed to promote Raf dimerization and hence its activation [51,52]. As shown in the present work, CaM would also attach to the region of α-helixes 4 and 5 of the globular domain of K-Ras. Interestingly, this region has recently been described as relevant for proper K-Ras dimerization, as arginine 161 forms a salt bridge when forming the dimer [53]. As stated above, according to our model the region used by CaM to interact with K-Ras may overlap with the one used to form K-Ras dimers. Moreover, not only do these interactions share the surfaces by which they interact but also certain residues used in the interaction such as arginine 161. Thus, one can conclude that CaM’s interaction with K-Ras would most probably interfere with K-Ras dimerization, and consequently this would be another mechanism (besides inhibiting phosphorylation [21]) by which CaM is negatively regulating K-Ras-Raf-ERK signaling.

All in all, we can affirm that our simulations (and later experimental validation) propose a reliable model in which residues R135, R161 and R164 play a significant role in the interaction of the globular domain of K-Ras with CaM, while the polybasic domain of the HVR interacts with the acidic linker region of CaM.

## Methods

### K-Ras and CaM joining

The joining of the proteins was performed in several steps. In order to have a model to work with, we first examined the original structure or the NMR structure of CaM with the HIV-1 matrix peptide (PDB code 2MGU). The peptide presented a certain degree of homology with the HVR of K-Ras, and we took advantage of that fact by replacing the existing peptide with a fragment of K-Ras (residues 165 to 188). To this end, the peptide was replaced using the program modeller (https://salilab.org/modeller/). The best structure generated by modeller was selected to perform the following simulations.

The HIV-1 matrix peptide was replaced by the K-Ras peptide. However, the homology between the sequences did not match the real orientation of the interaction between K-Ras and CaM. Thus, once the HIV-1 matrix peptide was replaced by the K-Ras fragment, another program was developed to rotate it. This software creates a vector between two given atoms (one from the CaM and another from the K-Ras fragment) and increases the module (the distance between those atoms). Afterwards, it performs a rotation on its axis (rotating the whole K-Ras fragment) and decreases the module (diminishes the distance between the selected atoms). Even though several combinations were tried, the distances were finally set to 10 Å and 5Å and the rotation angle was fixed at 180°. The system was then minimized in a multi-step manner, applying the same restraints as in the simulations with K-Ras. The minimized complex was heated up to 300 k in a step wise manner, at a rate of 30 K every 20 ps. The protein backbone atoms were restrained with a force constant of 0.5 kcal/mol·Å. Additionally, 200 ps of simulation at constant pressure (NPT ensemble) were performed without any restraint in order to allow density equilibration. Then, a short MD simulation of 2 ns length within the NVT assembly was done to allow small structural readjustments.

The final structure after this process was used as a reference to add the full-length oncogenic K-Ras (mutation G12D) (PDB code 4DSN). The lacking residues (a majoritarian part of the globular domain, residues 1–164) were added by merging the two systems. This step was done by superimposing the residues 165 to 168 of K-Ras and removing the leftover atoms of the K-Ras peptide.

### System preparation and minimization

The final complex was placed in a cubic periodic box filled with TIP3P water molecules, imposing a minimal distance of 15 Å between the protein and the box walls. Water molecules closer than 2.2 Å were removed and neutralizing counter-ions (sodium ions) were added at positions of lowest electrostatic potential. Minimization was carried in a multistep procedure: 1) Full complex restraint, both K-Ras and CaM were restrained with a 10 kcal/mol Å constant, including GTP and calcium ions; 2) CaM and globular domain of K-Ras restricted while releasing the lateral chains of the HVR; 3) Release of the lateral chains of CaM; 4) Progressive release of the HVR and CaM by diminishing the restriction constants from 10 to 0.5 kcal/mol Å; 5) Progressive release of the globular domain of K-Ras (in the same way as with HVR and CaM); 6) Minimum restraint on all backbones (0.1 kcal/mol Å); 7) No restraints minimization. These minimizations were performed through 5000 steps of the conjugate gradient algorithm keeping fixed the selected parts of the system fixed with the indicated restriction constants, except for the last minimization, which was carried out for 10000 steps.

### cMD and sMD simulations

First, the cMD simulations were carried out. To do so, the minimized systems were heated up to 300 K in increments of 30 degrees per step of 20 ps. Afterwards, 200 ps of simulation at NPT ensemble were performed. Also, a short MD simulation of 2 ns length within the NVT assembly was carried out.

The MD simulations of the systems were performed in a multi-step procedure (each step of 10 ns). The temperature was regulated by using the Langevin thermostat with a collision frequency ɣ of 2.0 ps-1. All bonds involving hydrogen atoms were constrained to their equilibrium value using the SHAKE algorithm, allowing the use of a 2 fs integration time step in all of the simulations. Non-bonded interactions were truncated at a cut-off of 10 Å, and long range electrostatic interactions were treated with the particle-mesh Ewald method. A total of 6 molecular dynamics simulations using different sets of initial velocities aimed at providing a better sampling [54] were performed of at least 50 ns each one.

As for the sMD simulations, the initial coordinates were taken from the first 5 ns of the cMD simulation. A λ factor of 0.8 was applied, and a total of 4 simulations of at least 50 ns were produced.

### Binding energy calculation with MMPB/GBSA

In order to determine the binding energy between the proteins, the AmberTools module of AMBER was used with Molecular Mechanics Poisson Boltzmann (Generalized Born) Solvation Area. To perform the calculations, structures for the conformational ensemble were extracted from the MD trajectories. The water molecules and the counter-ions were removed to obtain the topology of the systems without solvent. The calculations were performed with the following parameters. GB: GB method 5, salt concentration 0M and surface tension value 0.005. PB: Cavity offset -0.920, cavity surften 0.00542, external dielectric constant 80.0, internal dielectric constant 1 and ionic strength 0M.

### Umbrella sampling (US)

CaM and K-Ras were moved closer with a restriction between their suspected regions of interaction. A mass center was created in the globular domain of K-Ras, selecting the alpha carbons of the residues 161 to 169, and another was created in one of the lobes of CaM, selecting the alpha carbons of the residues 29, 32, 48, 52, 63 and 67. These two mass centres were restrained to keep a certain distance, between 20 and 5 Å. A restriction constant of 5 kcal/mol Å was applied at higher or shorter distances so that the mass center would remain close to the specified separation; if the simulations tend to approach or separate the mass centers, the restriction constant corrects the deviation. The distance was analyzed every 5 frames to make sure it was maintained as stablished. The backbone of residues 161 to 169 of K-Ras were also restricted so as to maintain their mass center stable. Five nanoseconds of each distance were obtained, decreasing the distance by 0.5 Å at each step (obtaining a total of 30 steps). Afterwards, the last coordinates of the simulations between 5 and 12 Å were obtained and a cMD of 10 ns was performed for each structure, and the interaction stability was analyzed with MMPB/GBSA analysis.

### Surface Plasmon Resonance (SPR) analysis

Byotinilated CaM was used as the ligand and GST-K-Ras as the analyte. A Sensor Chip SA was used for these experiments. This chip has a matrix of carboxymethylated dextran pre-immobilized with streptavidin, which binds to Byotinilated CaM while K-Ras was injected as an analyte. The matrix was first activated and prepared as follows: all four channels of the chip were treated with 100 mM HCl, 50 mM NaOH, 0.1% SDS and then water at a flow rate of 100 μL/min: and the signal was normalized by injecting glycerol at 70% and then two cycles of running buffer (50 mM Tris-HCl, pH 7.6, 150 mM NaCl, 0.05% Tween 20, 2 mM MgCl_2_ and 1 mM CaCl_2_). Then the ligand was loaded: the first channel was loaded with biocytin alone (a biotin analogue), in order to have a negative control and being able to detect any unespecificities; the rest of channels were loaded with biotinylated CaM; the loading was performed at 25°C with a concentration of 20 μg/mL of biotinylated CaM solved in running buffer; the ligand was added at a constant flow rate of 5 μL/min for 20 seconds (until 1000 resonance units (RU) were reached); afterwards, 0.3μg/mL of biocytin in running buffer were injected to block the streptavidin molecules that had not bound to biotinylated CaM. Next, the analyte, the globular domain of K-Ras (whether the WT form or the mutated forms), was injected at a flow rate of 5 μL/min for 1 minute. The protein was allowed to dissociate for a minute. Samples were loaded at concentrations between 0.0625 μM and 1μM. Last, the chips were regenerated by injecting running buffer without MgCl_2_/CaCl_2_ and supplementing with 6 mM EDTA, to obtain the apo form of CaM and dissociating any remains of K-Ras. The regeneration step was performed for a minute at 30 μL/min.

The data obtained were analysed with the Scrubber software. Both the kinetics and the affinity were analysed: 1) after loading the data file, the baseline was fixed as 0 for each flow cell (channel); 2) the injection points were aligned for all the samples; 3) the region of the sensogram regarding the injection of the sample was selected and cropped; 4) the samples were blanked subtracting the flow cell 1, which has not biotinylated CaM but biocytin; 5) the spikes generated by the injection or buffer exchange (when injecting the regeneration solution) were removed; 6) the bound section was selected as the more stable section within the injection process, usually 10 seconds before the ending of the injection; 7) for the affinity analysis, the amount of bound protein at each concentration of the sample was analysed to determine the K_D_. At least 4 concentrations were injected in each run.

To obtain the purified globular domain of K-Ras, the cDNA of residues 1–166 of K-Ras4B (either WT or with mutations in 1 or 2 bases) were cloned into a pGEX-KG bacterial expression vector with a GST tag. BL21(DE3) cells were transformed with the plasmids. The expression of the protein was induced by adding 0.5mM IPTG to a 0.5 L culture of the transformed cells and incubating for 4 hours. Cells were harvested at 6000 r.p.m. for 10 minutes at 4°C. The supernatant was discarded and the cell pellets were stored at -80°C. After checking a proper protein expression, cell pellets were lysed with a lysis buffer (50 mM TrisHCl pH 7.5, 100mM NaCl, 1mM EDTA, 5mM MgCl_2_, 10% Glycerol, 1% Triton X-100, 0.5% Β-Mercaptoethanol). Cells were lysed for 20 minutes on ice and then sonicated for three minutes, also on ice. The lysates were centrifuged for 10 minutes at 14000 r.p.m. at 4°C. The supernatant was collected and added to 800 μL of glutathione-sepharose beads. The mixture was incubated for 1 hour at 4°C. The supernatant was removed by centrifugation (3 minutes 3000 r.p.m.) and the beads were washed with 20mL of lysis buffer four times and once with exchange buffer (Tris-HCl pH 7.4 10 mM, NaCl 150 mM, MgCl_2_ 2 mM, glycerol 10%, DTT 1 mM)). Afterwards, the beads were incubated with 1.6 mL of exchange buffer complemented with 1mM GTP, 10mM EDTA and 1mM DTT. The mix was incubated for 30 minutes at room temperature. Magnesium chloride was added until a final concentration of 15 mM was reached, and then the beads were washed with 15 mL of exchange buffer with 2mM of MgCl_2_. The resin was separated by centrifugation and 1 mL of lysis buffer supplemented with 20mM of glutathione was added. The supernatant was then collected by centrifugation. The products of the purification were confirmed by western blot.
